# O.4.2-2 Exercise and screentime; clusters in cohorts of Swedish adolescents with and without disabilities

**DOI:** 10.1093/eurpub/ckad133.177

**Published:** 2023-09-11

**Authors:** Jennifer Gothilander, Camilla Eriksson, Lena Almqvist, Johanna Fritz

**Affiliations:** Mälardalen University, Sweden; jennifer.gothilander@mdu.se; Mälardalen University, Sweden; jennifer.gothilander@mdu.se; Mälardalen University, Sweden; jennifer.gothilander@mdu.se; Mälardalen University, Sweden; jennifer.gothilander@mdu.se

## Abstract

**Purpose:**

Identify clusters of Swedish adolescents based on the combination of their exercise and screentime, and investigate if gender and disability are associated with clusters. This person-oriented study identifies clusters based on activities and can provide a complementary perspective to other descriptive studies.

**Methods:**

This cohort study used register data from 564 adolescents aged 12-18 years, collected in 2019. Adolescents provided data on their exercise in sports clubs, self-organized exercise, watching TV or movies, and playing video games. Cluster analyses were done separately on 12-14 and 15-18-year-olds. Regression analysis was used to examine if gender and disability are associated with clusters.

**Results:**

Identified clusters are *Exercising & no watching TV, Using screens, No sporting, Sporting & no self-organized exercising, No watching TV or exercising, Watching TV, Self-organized exercising & no sporting*, and *Exercising*. *A*dolescents in the cluster *Exercising* are characterized by exercising in both sports clubs and self-organized exercise. *Exercising* was the largest cluster in both age groups with 39% of adolescents aged 12-14 years and 34% of adolescents aged 15-18 years. Twelve percent of 12-14-year-old adolescents and 18% of 15-18-year-old adolescents were in clusters characterized by not exercising in either sports clubs or self-organized. Gender was not associated with any cluster, and having a disability was associated with the cluster *Watching TV* in the older cohort (13% of adolescents aged 15-18).

**Conclusions:**

The study provides insight into the exercise and screentime habits of Swedish adolescents. Gender does not play a significant role in the clusters, while having a disability is associated with certain screentime behaviors in older adolescents. Overall, this person-oriented approach can complement other descriptive studies and inform interventions aimed at promoting healthy habits among adolescents.

**Funding:**

Swedish Research Council (2018-05824_VR).

